# p-Chloroaniline – Derivation of a BAR: Assessment Values in Biological Material – Translation of the German version from 2026

**DOI:** 10.34865/bb10647e11_2or

**Published:** 2026-06-30

**Authors:** Isabell Schönrath, Gabriele Leng, Hans Drexler, Andrea Hartwig, MAK Commission

**Affiliations:** 1 Currenta GmbH & Co. OHG, CUR-SIT-ABG-GS-BLM – Institute for Biomonitoring 51368 Leverkusen Germany; 2 40699 Erkrath Germany; 3 Friedrich-Alexander-Universität Erlangen-Nürnberg, Institute and Outpatient Clinic of Occupational, Social, and Environmental Medicine Henkestraße 9–11 91054 Erlangen Germany; 4 Institute of Applied Biosciences, Department of Food Chemistry and Toxicology, Karlsruhe Institute of Technology (KIT) Adenauerring 20a, Building 50.41 76131 Karlsruhe Germany; 5 Permanent Senate Commission for the Investigation of Health Hazards of Chemical Compounds in the Work Area, Deutsche Forschungsgemeinschaft, Kennedyallee 40, 53175 Bonn, Germany. Further information: Permanent Senate Commission for the Investigation of Health Hazards of Chemical Compounds in the Work Area | DFG

**Keywords:** p-Chloroaniline, biological reference value, BAR, background exposure

## Abstract

The German Commission for the Investigation of Health Hazards of Chemical Compounds in the Work Area evaluated the data for 4-chloroaniline [106-47-8] to derive a biological reference value (BAR). p-Chloroaniline is an intermediate in organic synthesis and is used in the manufacture of pharmaceuticals, plant protection products and dyes. As a potent inducer of methaemoglobin formation, the hazards regarding its acute toxicity surpass the carcinogenic potential. Hence, several accidents with a consecutive cyanosis are reported. Besides the methaemoglobin level, two other, more specific biomarkers are known: p-chloroaniline set free from the haemoglobin adduct and p-chloroaniline in urine. p-Chloroaniline after hydrolysis represents the sum of all *N*-conjugated phase II metabolites which are then back-converted to their mother compound. Based on studies of the general population, a BAR of 1 µg p-chloroaniline (after hydrolysis)/l urine has been derived. The smoking status did not influence the urinary levels of p-chloroaniline.

**Table:** 

**BAR (2025)**	**1 µg p-chloroaniline (after hydrolysis)/l urine** Sampling time: end of exposure or end of shift

**MAK value**	**–**
Absorption through the skin (1990)	H
Sensitization (2008)	Sh
Carcinogenicity (1990)	Category 2
	
Synonyms	1-Amino-4-chlorobenzene
CAS number	106-47-8
Formula	C_6_H_6_ClN
Molar mass	127.57 g/mol
Melting point	70 °C (IFA 2025)
Boiling point	232 °C (IFA 2025)
Relative density at 20 °C	1.43 g/cm^3^ (IFA 2025)
log K_OW_	1.83 (IFA 2025)

p-Chloroaniline is an intermediate in organic synthesis and is used in the manufacture of pharmaceuticals, plant protecting agents and dyes.

## Metabolism and Toxicokinetics

### Absorption, distribution and elimination

p-Chloroaniline can be absorbed by inhalation and through the skin (translated in Henschler 1992). Absorption through the skin, as described in the accidents discussed below (Herber 2024; Messmer et al. 2015; Pizon et al. 2009), can lead to significant methaemoglobin concentrations in humans. p-Chloroaniline penetrates the skin and has therefore been designated with an “H” (for substances which can be absorbed through the skin in toxicologically relevant amounts) (Henschler 1992). Test results for contact sensitization in guinea pigs and mice were positive. p-Chloroaniline has therefore been designated with “Sh” (for substances which cause sensitization of the skin) (Hartwig 2009).

### Metabolism

**Fig. 1:**
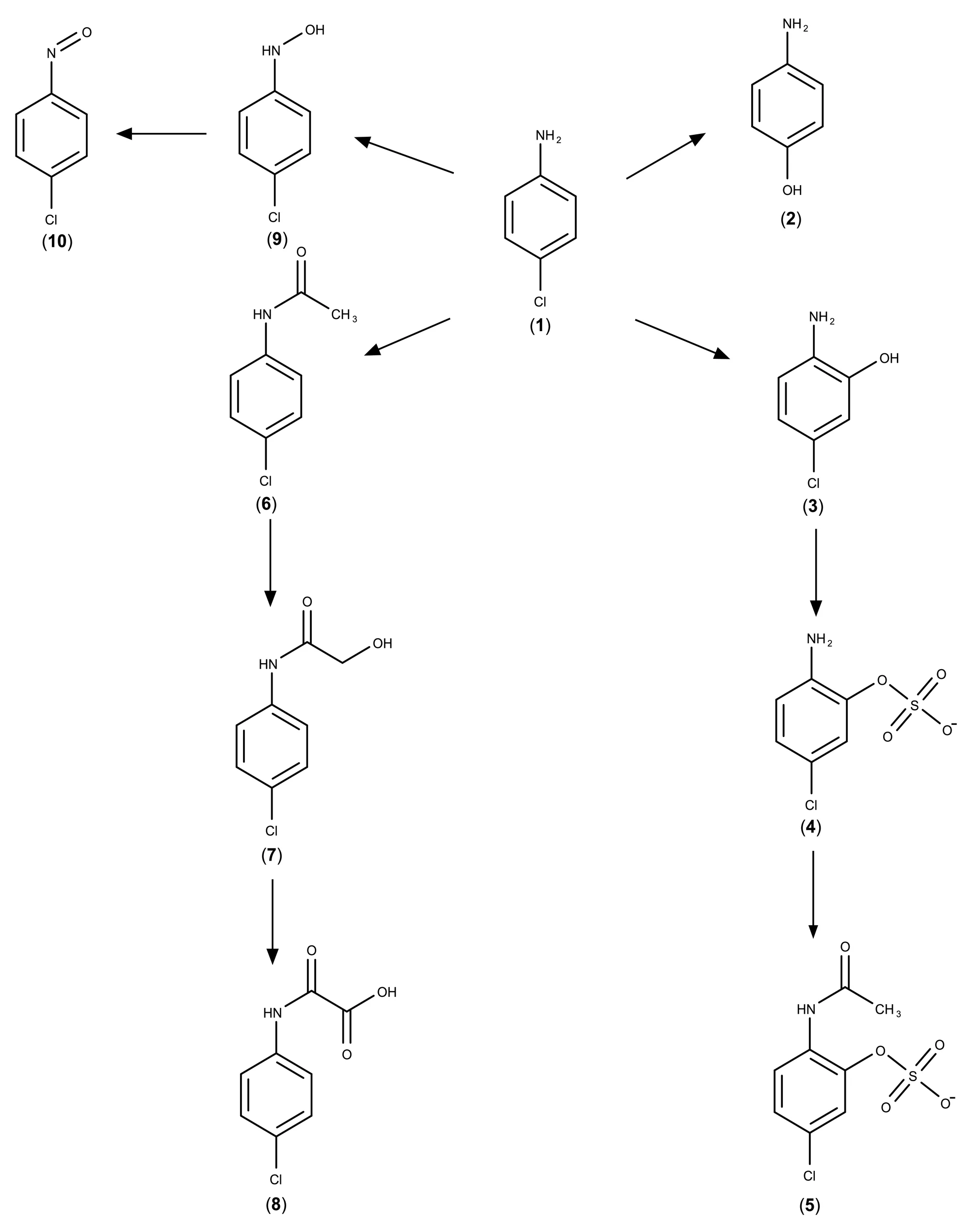
Metabolism of p-chloroaniline, adapted from Concise International Chemical Assessment Document 48: 4-chloroaniline (WHO 2003). WHO is not responsible for the content or accuracy of this adaptation.

The metabolism of p-chloroaniline has been studied in laboratory animals. Its pathways are similar to those in humans and have been confirmed in cases of acute poisoning in humans (WHO 2003). After ingestion, p-chloroaniline is metabolised in the liver to ring- and *N*-hydroxylated compounds and their conjugates. The four metabolic pathways are shown in Figure 1. The aromatic ring can be hydroxylated either at the para position with replacement of the chloride ion (2) or at the ortho position (3). In the latter case, the hydroxyl group is subsequently conjugated with a sulphate anion (4) and the amino group is acetylated (5). *N*-acetylation of p-chloroaniline produces 4-chloroacetanilide (6). The acetylation rate in humans depends on the individual acetylator status. In a further reaction, 4-chloroacetanilide (6) is converted to 4-chloroglycolanilide (7), which is further oxidised to an oxamic acid derivative (8). The particularly reactive compound 4-chloronitrosobenzene (10) is formed when p-chloroaniline is hydroxylated at the amino group (9) and subsequently oxidised. While metabolites (2), (5) and (8) have been detected in urine, the nitroso species (10) reacts with haemoglobin and is therefore also responsible for methaemoglobin formation. Further information on the metabolism and toxicokinetics of p-chloroaniline can be found in the WHO documentation (2003).

(1) p-chloroaniline, (2) 4-hydroxyaniline, (3) 2-hydroxy-4-chloroaniline, (4) 2-amino-5-chlorophenyl sulphate, (5) *N*-acetyl-2-amino-5-chlorophenyl sulphate, (6) 4-chloro-*N*-acetanilide, (7) 4-chloroglycolanilide, (8) *N*-(4-chlorophenyl)oxamic acid, (9) 4-chloro-*N*-hydroxyaniline, (10) 4-chloro­nitrosobenzene.

## Critical Toxicity

Compared with aniline, p-chloroaniline with a haemoglobin binding index of 569 is 25 times more potent than aniline, which has a haemoglobin binding index of 22 (Sabbioni 1994). Acute poisoning leads to cyanosis (Herber 2024). In addition to acute poisoning at work (Herber 2024; Messmer et al. 2015; Pizon et al. 2009), there have also been reports of premature babies with severe methaemoglobinaemia whose incubators were accidentally cleaned with chlorohexidine solution instead of distilled water. Chlorohexidine can spontaneously degrade into p-chloroaniline (van der Vorst et al. 1990). Methaemoglobin formation is discussed as the cause of the carcinogenicity of p-chloroaniline, as the breakdown of the no longer intact erythrocytes damages the spleen. Further information on its toxicity can be found in the MAK documentation (Henschler 1992).

## Exposure and Effects

From accidental exposures, correlations between 4-chloroaniline in urine or the haemoglobin adduct and the methaemoglobin formed were derived as health effect marker. However, the exact level of exposure to p-chloroaniline in these studies is unknown. The study of Lewalter and Korallus (1985) shows the exact time course of the methaemoglobin formation in the blood and the determined concentrations of p-chloroaniline in urine and as haemoglobin adduct after an accident that was not described in detail. The methaemoglobin level in the blood rose to 43.9% three hours after the accident. The peak of the p-chloroaniline concentration in urine occurred 30 minutes after the accident and then declined steadily. After three days, no p-chloroaniline could be detected. The concentration of p-chloroaniline in blood released from the haemoglobin adduct built up over the first three hours after the accident and was eliminated much more slowly, so that it was still detectable in blood one week after the accident (see Table 1; Lewalter and Korallus 1985). Even though the accident involved mixed exposure to aniline and p-chloroaniline, it can be assumed that p-chloroaniline played the major role in methaemoglobin formation due to its higher haemoglobin binding index. In another case, a worker was contaminated while handling waste containing p-chloroaniline; he had a methaemoglobin level of 69%. p-Chloroaniline was identified in his urine. However, p-chloroaniline analysis was only qualitative (Pizon et al. 2009). A worker who was exposed to p-chloroaniline dermally during cleaning work and possibly also by inhalation due to a defective breathing mask had a methaemoglobin level of 42.8%. Biomonitoring for p-chloroaniline metabolites in blood and urine was not performed (Messmer et al. 2015). After skin contact with p-chloroaniline and the onset of cyanosis, a methaemoglobin level of 18% and 161 µg p-chloroaniline/l blood set free from the haemoglobin adduct was detected in a worker in another case (see Table 1; Herber 2024).

**Tab. 1:** Concentrations of methaemoglobin in blood and p-chloroaniline in urine and/or blood (from haemoglobin adduct) after accidents

**Time after accident**	**Met-Hb** **[%]**	**p-Chloroaniline in urine** **[µg/g creatinine]**	**p-Chloroaniline as Hb adduct** **[µg/l blood]**	**References**
30 min	36.2*	1500	100	Lewalter and Korallus 1985
3 h	43.9*	500	300	
7 h	15.9*	200	200	
16 h	1.2*	50	100	
3 d	0.3*	< 10	100	
7 d	1,0*	< 10	50	
12 d	0.9*	< 10	< 10	
–	18	–	161	Herber 2024

^*^additional exposure to aniline

d: days; h: hours; Hb: haemoglobin; Met-Hb: methaemoglobin; min: minutes

## Selection of the Indicators

Exposure to p-chloroaniline can be detected in both urine and blood. In urine, hydrolysis is typically performed during sample preparation, converting phase II conjugates of p-chloroaniline into free p-chloroaniline. This allows both free and bound p-chloroaniline to be detected. It is no longer possible to distinguish between free and bound p-chloroaniline after hydrolysis.

In addition, haemoglobin adducts of p-chloroaniline can be determined. This parameter is based on the fact that aromatic amines are converted into reactive nitroso species and then react with a cysteine thiol group of haemoglobin. The adducts thus formed are stable throughout the entire lifespan of the erythrocyte (approx. 120 days) but can be hydrolysed in vitro under mild conditions.

Furthermore, methaemoglobin can be determined as a biological effect parameter. While a healthy person has only about 1% methaemoglobin (Mansouri and Lurie 1993), this value increases sharply when exposed to p-chloroaniline. For example, after occupational accidents involving p-chloroaniline, methaemoglobin levels of 69% were found after inhalation exposure (Pizon et al. 2009) and 42.8% after suspected dermal exposure (Messmer et al. 2015). As a biomarker, methaemoglobin has the disadvantage of low specificity for p-chloroaniline. The reason for this is that besides aromatic amines, various other substances, such as nitrites and chlorates, can cause methaemoglobin formation.

## Analytical Methods

In most cases, p-chloroaniline in urine is determined by gas chromatography after hydrolysis. The method most commonly used in German-speaking countries is probably the capillary gas chromatography and mass-selective detection in the single ion monitoring mode (GC-MSD in SIM mode; Weiss and Angerer 2002). An older method using GC-ECD (electron capture detector) was published by Riffelmann et al. (1995). In addition, liquid chromatographic methods with UV detection (Below et al. 2004; Jones et al. 2007) or EC detection (Lores et al. 1980) and, in more recent studies, also with mass-selective detectors are known (Chinthakindi and Kannan 2021).

The determination of p-chloroaniline, which is hydrolysed from the haemoglobin adduct, is described in a DFG method using GC-MS with negative chemical ionization (translated in Lewalter et al. 2001).

## Background Exposure

In a study by Riffelmann et al. (1995), exposure to various aromatic amines, including p-chloroaniline, was compared between potentially exposed workers and a control group. Both, the concentration of p-chloroaniline in urine and the concentration of p-chloroaniline in blood released from the haemoglobin adduct, were determined. The control group consisted of 16 men (8 smokers, 8 non-smokers). Only the measurements of p-chloroaniline in urine are reported below: no p-chloroaniline was found in the eight non-smokers, whereas p-chloroaniline was detected in less than half of the eight smokers. The maximum concentration was 0.8 µg p-chloroaniline/l urine.

Weiß and Angerer examined the urine of 20 individuals (gender unknown) for p-chloroaniline. With a detection limit of 0.05 µg/l, the detection rate was 90%. The median was 0.11 µg/l and the 95^th^ percentile was 0.57 µg p-chloroaniline/l urine. At 1.1 µg p-chloroaniline/l, the maximum value was almost twice as high as the 95^th^ percentile (Weiss and Angerer 2002). In his dissertation, Weiß examined the exposure of 197 test persons (gender unknown) from the general population to various aromatic amines, including p-chloroaniline. With a detection limit of 0.05 µg p-chloroaniline/l urine, the detection rate was 85.8%. The median was 0.185 µg/l and the 95^th^ percentile was 1.1 µg p-chloroaniline/l (Weiß 2005).

The most comprehensive population study to date on exposure to aromatic amines was conducted by Kütting et al. (2009) with 911 men and women (15–84 years) and 93 children (< 15 years) from Bavaria. Here, the median concentration in urine was 0.03 µg p-chloroaniline/l, which is below the limit of quantification. The 95^th^ percentile was 0.93 µg p-chloroaniline/l urine. The total collective was divided into smokers (n = 145) and non-smokers (n = 856) in order to investigate the influence of smoking status on p-chloroaniline exposure. The median urine level was also below the limit of quantification for both sub-collectives. The 95^th^ percentile and the maximum value for smokers were 0.61 µg/l and 3.02 µg p-chloroaniline/l urine versus 0.98 µg/l and 42.13 µg p-chloroaniline/l urine for non-smokers, respectively (Kütting et al. 2009). The discrepancy between the values is most likely due to a distortion caused by the large difference in the size of the collectives. In any case, there is no apparent influence of smoking status.

In a study examining 15 US test persons (7 men and 8 women, 10 Asians and 5 Caucasians; 213 urine samples (10–16 per test person)), the detection rate was 67.7% at a detection limit of 0.05 µg/l. The median was 0.085 µg/l urine or 0.051 µg p-chloroaniline/g creatinine and the 95^th^ percentile was 1.326 µg/l urine or 1.238 µg p-chloroaniline/g creatinine. The maximum values were 3.84 µg/l urine or 3.05 µg p-chloroaniline/g creatinine (Chinthakindi and Kannan 2022).

The data on background levels are presented in Table 2.

**Tab. 2:** p-Chloroaniline in the urine of the general population not occupationally exposed to p-chloroaniline.

Collective	% > LOD/LOQ	p-Chloroaniline in urine [µg/l urine] (µg/g creatinine)					References
		Mean value	Median	95^th^ Perc.	Range	LOD/LOQ	
**Germany**														
8 smokers	–	0.1	< LOD	–	< LOD–0.8	1/–	Riffelmann et al. 1995
8 non-smokers	–	< LOD	< LOD	< LOD	< LOD	1/–	
197	85.8	–	0.185	1.101	< LOD–39.1	0.05/–	Weiß 2005
98, urban population	81.6	–	0.184	1.219	< LOD–2.562	0.05/–	
99, rural population	89.9	–	0.186	1.015	< LOD–39.1	0.05/–	
20	90	–	0.11	0.57	< LOD–1.1	0.05/–	Weiss and Angerer 2002
1004	38.2	0.31	< LOQ	0.93	< LOQ–42.13	–/0.03	Kütting et al. 2009
145 smokers	34.5	0.15	< LOQ	0.61	< LOQ–3.02	–/0.03	
856 non-smokers	38.5	0.33	< LOQ	0.98	< LOQ–42.13	–/0.03	
**USA**														
n = 15, 213 samples (several samples per person)	67.7	0.25 (0.17)	0.085 (0.051)	1.326 (1.238)	< LOD–3.84	0.05/–	Chinthakindi and Kannan 2022

LOD: Limit of detection; LOQ: Limit of quantification; Perc.: Percentile

## Evaluation

Since p-chloroaniline has been shown to be clearly carcinogenic in animal experiments and has been classified in Carcinogen Category 2, a biological tolerance value (BAT value) has not been derived. The available data are insufficient to derive a biological guidance value (BLW). Due to the lack of data for both blood and urine, it is also not possible to derive exposure equivalents for carcinogenic substances (EKA). However, the data available for background exposure to p-chloroaniline in urine are sufficient to derive a biological reference value (BAR). The most important data for deriving the BAR come from the field study of 1004 people from Bavaria, which reported a 95^th^ percentile of 0.93 µg p-chloroaniline/l urine (Kütting et al. 2009). The 95^th^ percentiles of 0.57 µg/l (Weiss and Angerer 2002), 1.1 µg/l (Weiß 2005) and 1.326 µg/l (Chinthakindi and Kannan 2022) from the other studies were of the same order of magnitude and can therefore be regarded as confirmation of the results obtained by Kütting et al. (2009). Therefore, a

**BAR of 1 µg p-chloroaniline (after hydrolysis)/l urine**

is set. Sampling should be carried out after exposure or at the end of the shift. The possible influence of the smoking status on p-chloroaniline exposure discussed by Riffelmann et al. (1995) could not be observed in later studies, so that smoking cannot be assumed to have a relevant effect on the excretion of p-chloroaniline in urine. Since all studies except one reported the results of p-chloroaniline determination in relation to urinary volume, the BAR also uses volume rather than creatinine as a reference.

## Interpretation

The BAR refers to normally concentrated urine in which the creatinine content should be in the range of 0.3–3 g/l. As a rule, for urine samples outside the above-mentioned limits, it is recommended to repeat the measurement in the normally hydrated test person (translated in Bader et al. 2016).
